# Impact of Coexisting Coronary Artery Disease on the Occurrence of Cerebral Ischemic Lesions after Carotid Stenting

**DOI:** 10.1371/journal.pone.0094280

**Published:** 2014-04-14

**Authors:** Kuo-Lun Huang, Yeu-Jhy Chang, Chien-Hung Chang, Ting-Yu Chang, Chi-Hung Liu, I-Chang Hsieh, Ho-Fai Wong, Yau-Yau Wai, Yu-Wei Chen, Bak-Sau Yip, Tsong-Hai Lee

**Affiliations:** 1 Stroke Center and Department of Neurology, Linkou Chang Gung Memorial Hospital, Taoyuan, Taiwan; 2 College of Medicine, Chang Gung University, Taoyuan, Taiwan; 3 Department of Electrical Engineering, Chang Gung University, Taoyuan, Taiwan; 4 Department of Internal Medicine, Section of Cardiology, Linkou Chang Gung Memorial Hospital, Taoyuan, Taiwan; 5 Department of Medical Imaging and Intervention, Linkou Chang Gung Memorial Hospital, Taoyuan, Taiwan; 6 Department of Neurology, Landseed Hospital, Taoyuan, Taiwan; 7 Department of Neurology, National Taiwan University Hospital, Taipei City, Taiwan; 8 Department of Computer Science and Information Engineering, National Central University, Zhongli City, Taiwan; 9 Department of Neurology, National Taiwan University Hospital Hsinchu Branch, Hsinchu, Taiwan; University of Bologna, Italy

## Abstract

**Background:**

Coronary artery disease (CAD) may coexist with extracranial carotid artery stenosis (ECAS), but the influence of CAD on procedure-related complications after carotid artery stenting (CAS) has not been well investigated. The study aimed to determine the impact of CAD on the occurrence of peri-CAS cerebral ischemic lesions on diffusion-weighted imaging (DWI) scanning.

**Methods:**

Coronary angiography was performed within six months before CAS. DWI scanning was repetitively done within 1 week before and after CAS. Clinical outcome measures were stroke, angina, myocardial infarction and death within 30 days.

**Results:**

Among 126 patients (69.5±9.0 years) recruited for unilateral protected CAS, 33 (26%) patients had peri-CAS DWI-positive lesions. CAD was noted in 79% (26 in 33) and 48% (45 in 93) of patients with and without peri-CAS DWI-positive lesions (OR, 4.0; 95% CI, 1.6–10.0; *P* = .0018), and the number of concomitant CAD on coronary angiography was positively correlated with the risk for peri-CAS DWI-positive lesions (*P* = .0032). In patients with no CAD (n = 55), asymptomatic CAD (n = 41) and symptomatic CAD (n = 30), the occurrence rates of peri-CAS DWI-positive lesions were 13%, 41% and 30% (*P* = .0048), and the peri-CAS stroke rates were 2%, 7% and 0% (*P* = .2120).

**Conclusions:**

The severity of morphological CAD and the presence of either symptomatic or asymptomatic CAD are associated with the occurrence of peri-CAS cerebral ischemic lesions.

## Introduction

Extracranial carotid artery stenosis (ECAS) is a risk factor for cerebral ischemic stroke by causing cerebral embolism and cerebral hypoperfusion as well [1]. Carotid endarterectomy (CEA) is the current standard treatment for significant ECAS, and carotid artery stenting (CAS) is an alternative for high-risk patients, such as those with coronary artery disease (CAD), history of neck radiotherapy, and bilateral carotid artery stenosis [2]. Even though the long-term treatment outcome is similar between the two treatment choices [3], a recent randomized study has shown that there are more procedure-related cerebral ischemic lesions on diffusion-weighted imaging (DWI) scanning in patients receiving CAS than CEA [4].

Atherosclerosis is a chronic and multi-territory disease. In patients with ECAS, CAD is the most common concomitant vascular disease with the concurrence rate as high as 57% to 77% [5], [6], [7]. Although the presence of concomitant CAD has been shown to be associated with the long-term major cardiac events after CAS [8], there are limited studies investigating the impact of CAD on the occurrence of peri-CAS cerebral ischemic lesions. In a recent study by Chung *et al*, the presence of CAD was associated with increased amount of emboli production captured by embolic protection devices (EPDs) during CAS [9]. However, whether these emboli would lead to the occurrence of cerebral ischemic lesions or even stroke remains unanswered [10], [11]. Furthermore, only 39% to 59% of patients with concomitant ECAS and CAD had overt manifestations of myocardial ischemia [6], [7]. Therefore, patients with asymptomatic CAD may be unrecognized during the pre-CAS evaluation of stroke risk.

Considering the high prevalence rate of CAD in patients with ECAS, the present study aimed to determine whether the presence of morphological CAD on coronary angiography is a risk for peri-CAS cerebral ischemic lesions on DWI scanning, and also would like to investigate the influence of both asymptomatic and symptomatic CAD on the risk for peri-CAS cerebral DWI-positive lesions.

## Methods

### Subjects

**Figure 1:**
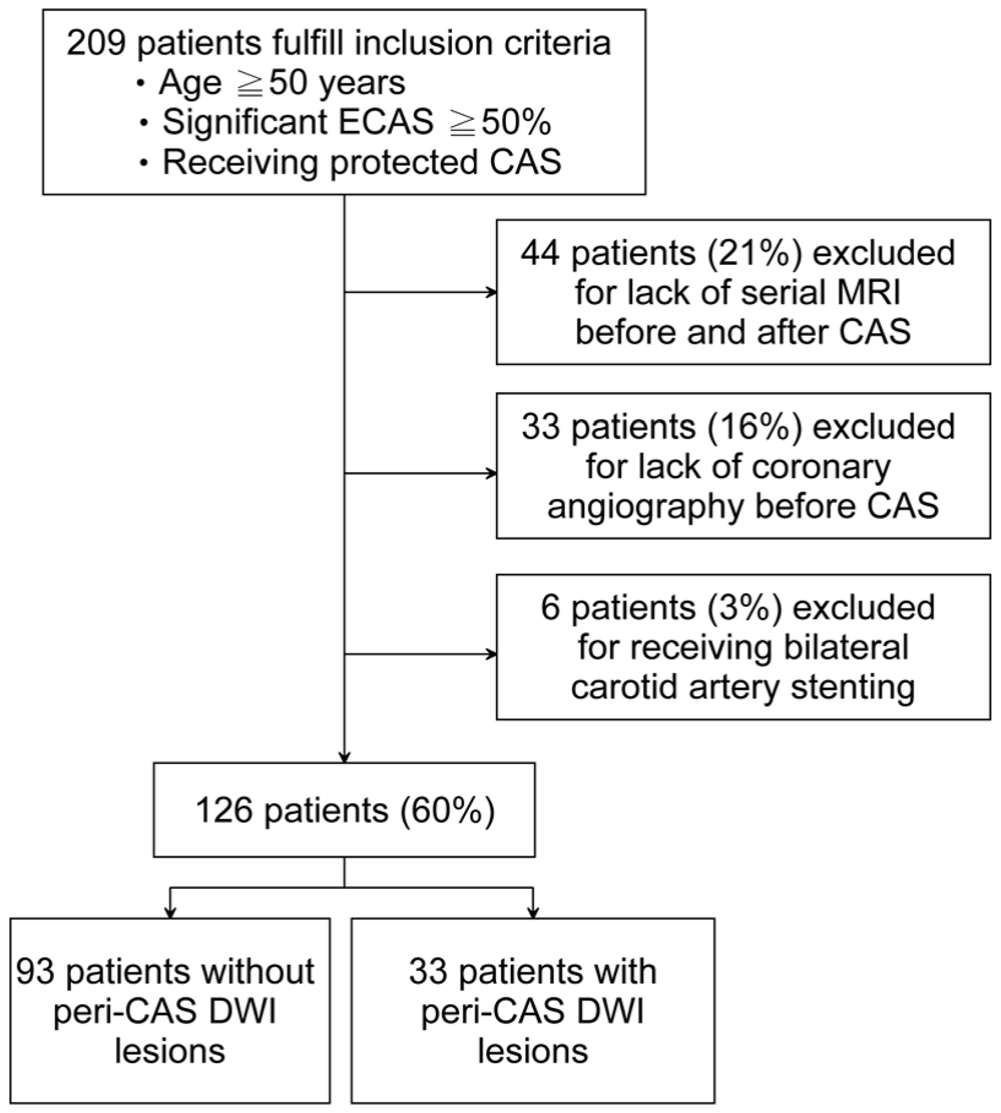
Flow chart for patient collection. The number of patients receiving unilateral protected CAS with coronary angiography and serial MRI evaluation (ECAS, extracranial carotid artery stenosis; CAS, carotid artery stenting; DWI, diffusion-weighted imaging; MRI, magnetic resonance imaging).

From December 2005 to December 2012, we included patients with 1) age ≧50 years, 2) significant ECAS ≧50% by NASCET criteria on cerebral angiography [12], and 3) receiving CAS with EPDs. The exclusion criteria were 1) lack of serial MRI scanning within 1 week before and within 1 week after CAS, 2) lack of coronary angiography study before CAS, or 3) receiving bilateral CAS. A total of 126 patients fulfilled the selection criteria (Figure 1), and there was no significant difference in demographic factors or ECAS severity between patients included and excluded. The patient data and procedure results were prospectively collected and retrospectively analyzed from our stroke registry databank [13]. All patients or next of kin gave written informed consent to angiography and MRI examinations and CAS treatment. To obtain clinical information from the stroke registry databank for analysis, patient records were anonymized and de-identified prior to data retrieval and analysis. The Institute Ethics Committee of Chang Gung Memorial Hospital approved the study protocol and data retrieval process.

### Evaluation of coronary artery disease and concomitant stenosis in other arteries

Coronary angiography and aortogram were reviewed for the study if they were performed within six months before or simultaneously with cerebral angiography prior to CAS [8]. Coronary artery disease and stenosis of extracranial vertebral artery, subclavian artery and iliac artery were defined as having artery diameter narrowing ≧50% in the projection showing the greatest severity [5]. Patients having evidence of CAD on coronary angiography were further divided into asymptomatic and symptomatic CAD by history of angina, myocardial infarction, or coronary revascularization in medical records [14].

### Carotid artery stenting procedure

Carotid artery stenting was performed with WALLSTENT (Boston Scientific Corp, Natick, MA) or Zilver stent (Cook Medical, Bloomington, IN). EPI FilterWire (Boston Scientific Corp, Natick, MA), post-stenting angioplasty with balloon dilation and intra-procedural heparinization were applied in all patients. A combination of aspirin (100 mg/day) and clopidogrel (75 mg/day) was routinely administered 72 hours before CAS, and clopidogrel was prescribed for 3 months and aspirin was maintained indefinitely after CAS. Patients were evaluated 1 day before and 1 day and 30 days after CAS by independent neurologists [15].

### MRI studies

Brain MRI was repeatedly performed within 1 week before and after CAS. MRI was obtained at a 1.5 Tesla scanner (Symphony, Siemens) with 5-mm slice thickness and 0.5-mm interslice gap for all sequences, including axial T1-weighted (T1W) sequence (spin-echo; repetition time [TR]/echo time [TE] = 449/12 ms; matrix 256×256), axial T2-weighted (T2W) sequence (fast spin-echo; TR/TE = 4000/90), fluid-attenuated inversion recovery sequence (TR/TE/TI = 9416/90/2200), and DWI sequence (single-shot spin-echo echo-planar technique; TR/TE = 2812/60; b values of 0, 500, and 1000 s/mm^2^). Peri-CAS acute ischemic events were defined as the occurrence of hyperintensity lesions on the post-CAS DWI scanning which were not observed in the pre-CAS MRI studies [15].

### Statistical analysis

All variables were summated using mean ± standard deviation for continuous variables and frequency distribution for categorical variables. Comparisons between groups were conducted with the Student's t test or analysis of variance (ANOVA) with Bonferroni adjustment where appropriate for continuous variables, and the chi-square test or Fisher's exact test for categorical variables. Logistic regression method with cumulative logit modeling for ordinal response variables was applied to obtain odds ratio (OR) and 95% confidence interval (CI) when response variables had more than 2 categories, and it was also applied to adjust for confounding factors [15]. SAS version 9.0 or higher was used for analyses, and p<0.05 was considered significant.

## Results

### Patient and procedural data

The study recruited 126 patients receiving unilateral protected CAS. The mean age of patients was 69.5±9.0 years, and 112 of them (89%) were men. The stenosis severity values of the treated and non-treated carotid arteries were 76.1±11.2% and 35.1±32%, respectively, and 47 of the 126 patients (37%) had symptomatic carotid stenosis. Of the 126 carotid artery stents, 122 were WALLSTENT and the rest were Zilver stent. EPI FilterWire was attempted in all patients except one treated carotid artery due to difficulty in anatomical approach. Within 30 days after CAS, 4 patients had minor ischemic stroke with the increase of NIH stroke scale <4, and no peri-procedural myocardial infarction or mortality was observed.

Thirty three of the 126 patients (26%) had new cerebral DWI-positive lesions on DWI scanning after CAS. Comparing to patients without peri-CAS DWI-positive lesions, patients with DWI-positive lesions had a higher frequency of concomitant CAD on coronary angiography (OR, 4.0; 95% CI, 1.6–10.0; *P* = .0018), but there was no difference in conventional vascular risk factors or ECAS severity between the two groups (*P*>.05, Table 1). The frequencies of concomitant arterial stenosis in vertebral artery, iliac artery and subclavian artery were 44%, 22% and 13%, and their presence was not related to the occurrence of peri-CAS DWI-positive lesions.

**Table 1:** Baseline characteristics of patients according to the occurrence of peri-CAS DWI-positive lesions.

	Without DWI-positive lesions	With DWI-positive lesions	*P*
N	93	33	
Continuous factors, mean (SD)*			
Age, years	69.3 (9.5)	69.8 (7.5)	0.7915
Stenosis severity of treated carotid arteries, %	75.7 (11.7)	77.1 (10.0)	0.5401
Stenosis severity of non-treated carotid arteries, %	35.4 (32.0)	34.3 (32.6)	0.8665
Left ventricular ejection fraction, %	64.3 (11.3)	65.7 (12.3)	0.5610
Serum creatinine, mg/dL	1.01 (0.28)	1.07 (0.34)	0.3740
Categorical factors, N (%)†			
Gender, male	82 (88)	30 (91)	1.0000
Symptomatic carotid artery stenosis	35 (38)	12 (36)	0.8967
Diabetes mellitus	32 (34)	14 (42)	0.4142
Hypertension	75 (81)	28 (85)	0.5854
Dyslipidemia	67 (72)	22 (67)	0.5632
Findings on coronary angiography or aortogram			
Concomitant CAD	45 (48)	26 (79)	0.0018‡
Concomitant vertebral artery stenosis	41 (44)	15 (45)	0.8919
Concomitant iliac artery stenosis	19 (20)	9 (27)	0.4237
Concomitant subclavian artery stenosis	12 (13)	4 (12)	1.0000
Peri-CAS manifesting stroke	0 (0)	4 (12)	0.0041
History of anti-platelet medication over 90 days before CAS	56 (60)	22 (67)	0.5095

*Comparison with two-group t test.

† Comparison with chi-square test or Fisher's exact test.

‡ p<0.01 after adjustment for age, gender, LVEF, creatinine level, symptomatic carotid stenosis and anti-platelet medication history.

CAS, carotid artery stenting; DWI, diffusion-weighted imaging; CAD, coronary artery disease.

### Relationship between morphological CAD and peri-CAS ischemic lesions

In the 55 patients without CAD, there were 7 having peri-CAS DWI lesions and 1 having peri-CAS stroke, and in the 71 patients with CAD, there were 26 having peri-CAS DWI lesions (OR, 4.0; 95% CI, 1.6–10.0; *P* = .0018) and 3 having peri-CAS stroke (OR, 2.4; 95% CI, 0.2–23.6; *P* = .6313). In patients with 0-vessel, 1-vessel, 2-vessel and 3-vessel CAD, the frequencies of peri-CAS DWI-positive lesions were 13%, 23%, 52% and 33% (*P* = .0032; 1-vessel versus 0-vessel OR, 2.1 with 95% CI, 0.7–6.7; 2-vessel versus 0-vessel OR, 7.3 with 95% CI, 2.5–21.6; 3-vessel versus 0-vessel OR, 3.4 with 95% CI, 0.8–11.4), and the frequencies of peri-CAS manifesting stroke were 2%, 3%, 7% and 0% (*P* = .6470; OR is not provided because the maximum likelihood estimate may not exist), respectively.

### Relationship between symptomatology of CAD and peri-CAS ischemic lesions

Seventy one patients (56%) had concomitant CAD on coronary angiography, and 41 of them (58%) were asymptomatic CAD. Considering the composite findings on coronary angiography and the relevant cardiac symptoms and signs, there were 55 patients without CAD (Group I), 41 patients with asymptomatic CAD (Group II), and 30 patients with symptomatic CAD (Group III) (Table 2). In Group III, there were 27 patients with angina, 9 with myocardial infarction, 17 with prior coronary revascularization, 9 with ischemic changes on echocardiography, 10 with ischemic changes on treadmill or thallium stress tests, and 4 with congestive heart failure of New York Function Class III or IV. In Group I, there were 8 patients with previous neck radiation therapy, 10 with contralateral carotid artery stenosis >70%, and 5 with age >80 years. The rest patients of Group I preferred CAS to CEA even after well explanation of the benefits and risks between the two interventional choices.

**Table 2:** Clinical and imaging characteristics of patients with no CAD, asymptomatic CAD and symptomatic CAD.

	No CAD, Group I	Asymptomatic CAD, Group II	Symptomatic CAD, Group III	*P*
N	55	41	30	
Continuous factors, mean (SD)*				
Age, years	67.5 (9.4)	70.4 (8.9)	71.9 (7.8)	0.0722
Stenosis severity of treated carotid arteries, %	75.7 (12.0)	76.8 (10.5)	75.6 (11.2)	0.8812
Stenosis severity of non-treated carotid arteries, %	33.3 (32.8)	32.6 (28.7)	42.0 (34.7)	0.4093
Left ventricular ejection fraction, %	67.0 (8.8)	65.3 (12.6)	59.4 (13.0)‡	0.0127
Serum creatinine, mg/dL	0.95 (0.25)	1.08 (0.34)	1.09 (0.29)	0.0294
Categorical factors, N (%)†				
Gender, male	48 (87)	37 (90)	27 (90)	0.9360
Symptomatic carotid artery stenosis	24 (44)	17 (41)	6 (20)	0.0658
Diabetes mellitus	16 (29)	15 (37)	15 (50)	0.1638
Hypertension	40 (73)	36 (88)	27 (90)	0.0675
Dyslipidemia	38 (69)	24 (59)	27 (90)	0.0091
Atrial fibrillation	3 (5)	2 (5)	4 (13)	0.3738
Number of CAD on coronary angiography				<0.0001§
No CAD	–	–	–	
1-vessel CAD	–	26 (64)	4 (13)	
2-vessel CAD	–	12 (29)	17 (57)	
3-vessel CAD	–	3 (7)	9 (30)	
Peri-CAS cerebral DWI-positive lesions	7 (13)	17 (41)	9 (30)	0.0048∥
Peri-CAS manifesting stroke	1 (2)	3 (7)	0 (0)	0.2120
History of anti-platelet medication over 90 days before CAS	25 (45)	23 (56)	30 (100)	<0.0001

*Comparison with ANOVA test.

† Comparison with chi-square test or Fisher's exact test.

‡ p<0.05 versus patients with no CAD by Bonferroni post hoc analysis.

§ Comparison between patients with asymptomatic and symptomatic CAD.

∥ p<0.01 after adjustment for age, gender, LVEF, creatinine level, symptomatic carotid stenosis, hypertension, dyslipidemia and anti-platelet medication history.

CAS, carotid artery stenting; DWI, diffusion-weighted imaging; CAD, coronary artery disease.

Peri-CAS DWI-positive lesions were found in 13%, 41%, and 30% of patients (*P* = .0048; Group II versus Group I OR, 4.9 with 95% CI, 1.8–13.3; Group III versus Group I OR, 2.9 with 95% CI, 1.0–8.9), and peri-CAS manifesting stroke was found in 2%, 7%, and 0% of patients from Groups I to III, respectively (*P* = .212; OR is not provided because the maximum likelihood estimate may not exist). Patients with symptomatic CAD (Group III) had tendency toward older age (*P* = .0722), worse left ventricular ejection fraction (LVEF) (*P* = .0127) and creatinine (*P* = .0294), a lower percentage of symptomatic ECAS (*P* = .0658; Group II versus Group I OR, 0.92 with 95% CI, 0.40–2.07; Group III versus Group I OR, 0.32 with 95% CI, 0.11–0.92), and higher percentages of hypertension (*P* = .0675; Group II versus Group I OR, 2.70 with 95% CI, 0.89–8.17; Group III versus Group I OR, 3.38 with 95% CI, 0.89–12.79), dyslipidemia (*P* = .0091; Group II versus Group I OR, 0.63 with 95% CI, 0.27–1.47; Group III versus Group I OR, 4.03 with 95% CI, 1.07–15.12) and anti-platelet medication over 90 days before CAS (*P*<.0001; OR is not provided because the maximum likelihood estimate may not exist) than the other two groups, and they also had greater number of diseased coronary artery than patients with asymptomatic CAD (*P* = <.0001). After adjusting for age, gender, LVEF, creatinine level, symptomatic carotid stenosis, hypertension, dyslipidemia and anti-platelet medication history, patients with either asymptomatic (OR, 4.9; 95% CI, 1.7–14.0; *P* = .0031) or symptomatic CAD (OR, 3.5; 95% CI, 0.9–12.9; *P* = .0639) had a moderate to significant risk for the occurrence of peri-CAS DWI-positive lesions comparing to patients with no CAD. Among patients with CAD (Groups II & III), the history of taking anti-platelet medication over 90 days was associated with the presence of symptomatic ECAS (OR, 0.26; 95% CI, 0.08–0.80; *P* = .0175).

## Discussion

Coronary artery disease is the most common concomitant vascular disease in patients with ECAS, and their coexistence has been shown to be associated with carotid plaque composition and characteristics of peri-CAS emboli captured on EPDs [9]. Our previous report has noticed that the presence of CAD might influence the occurrence of peri-CAS silent cerebral ischemic lesions on DWI scanning [15]. However, most of the studies diagnosed CAD by clinical history and did not examine the coronary angiographic findings [9], [16], [17]. Furthermore, it is not studied if symptomatic and asymptomatic CAD would carry a similar risk for peri-CAS DWI-positive lesions, as asymptomatic CAD comprises a substantial proportion of patients with ECAS [7], [18]. In the present series, the diagnosis of CAD, either symptomatic or asymptomatic, was documented on coronary angiography before undertaking protected CAS. Our study demonstrated that the risk for peri-CAS cerebral DWI-positive lesions was higher in patients with CAD, especially those with asymptomatic CAD.

CAS is preferred to CEA in patients with significant ECAS when there is concomitant CAD. On the other hand, the presence of CAD is also regarded as a major risk factor for peri-operative stroke, but only limited studies focused on the influence of CAD on the peri-CAS cardiac and neurological risks [8], [19]. Although the presence of CAD on angiography is reported to be associated with long-term cardiac and all-cause mortality [8], our study showed the 30-day risk for myocardial infarction or angina after CAS was relatively low, which is concordant with the recent report [19]. In our study, the frequencies of concomitant stenosis in coronary artery, vertebral artery, iliac artery and subclavian artery were 56%, 44%, 22% and 13% respectively, and the frequency order was compatible with Wu *et al*'s report [7]. On the other hand, the non-treated carotid artery stenosis severity and the frequencies of concomitant stenosis in other arteries were similar between patients with and without CAD (Table S1). As to the relationship between peri-CAS cerebral emboli and CAD, Chung *et al* reported that patients with prior myocardial infarction had a greater number of peri-CAS embolic particles with smaller minimum size (109±71 µm in diameter) than patients without prior myocardial infarction (212±200 µm in diameter) [9], and debris particles smaller than the filter pores of the EPDs may have the chance to pass through embolic protection devices [20]. However, the association between the coexisting CAD and the occurrence of peri-CAS cerebral ischemic lesions was not investigated. In our study, only the concomitant CAD imposed adverse effects on the occurrence of peri-CAS ischemic lesions (Table 1). Such findings may suggest that the higher risk for peri-CAS DWI-positive lesions in CAD patients can possibly be related to the fact that CAD is a marker of more diffuse atherosclerosis.

Both CAD and ECAS are the consequence of systematic atherosclerosis. Systematic inflammation may destabilize carotid plaques in patients with unstable angina [21], and administration of low-dose aspirin over 3 months may be capable of stabilizing carotid plaques through its anti-inflammatory and anti-atherogenic effects [22]. In our study, patients with symptomatic CAD had a higher percentage of taking anti-platelet medication than asymptomatic CAD, and the history of taking anti-platelet medication over 90 days was associated with the presence of symptomatic ECAS (*P* = .0175) in patients with CAD. It is speculated that carotid plaques could be more labile in patients without anti-platelet medication, and that may in part explain why asymptomatic CAD had a higher risk for peri-CAS cerebral vascular events in our study.

Although the presence of CAD is one of the indications in choosing CAS for carotid revascularization [23], we found that it should be more precautious when performing CAS in patients with coexisting CAD since they may also have a higher risk for peri-CAS cerebral ischemic lesions comparing to those without coexisting CAD. However, the optimal pre-operation survey for CAD remains a debate in patients with ECAS. Although both our and the previous studies showed that simultaneous diagnostic coronary angiography with CAS carried little additional risk for procedure-related complications [6], the invasive characteristics of coronary angiography limit its widespread application for the CAD survey in routine clinical practice. Several non-invasive imaging modalities, including multi-detector computed tomography (MDCT) angiography and contrast-enhanced cardiac MRI, have been proposed to evaluate the presence of CAD in patients with ECAS [18], [24]. In a recent study comparing the diagnostic performance for detecting CAD between the coronary MDCT and MR angiography based on the findings of the coronary conventional angiography, Liu *et al* found coronary MR angiography has favorable ability in detecting significant stenosis than MDCT angiography, with the sensitivity and specificity of 75∼83% and 71∼81% respectively [25]. Such finding suggests that the non-invasive imaging survey for the presence of CAD is feasible and may be helpful for stratifying the risk of peri-CAS cerebral ischemic lesions.

The application of EPDs in patients receiving CAS has been reported to reduce the occurrence of procedure-related stroke [26], but there are still a certain proportion of patients, from 17% to 73%, having silent cerebral DWI-positive lesions after protected CAS [4], [11]. In our study, only one type of protection device (FilterWire) was used in all patients, which can avoid the variation in protection effect of different filter types. The occurrence rate of cerebral DWI-positive lesions after protected CAS was 26% (33 in 126 patients), and it was similar to previous reports. Although most peri-CAS DWI-positive lesions are clinically silent [27], they may impose adverse effects on the cognitive function after carotid revascularization [15], [28]. Therefore, patients having peri-CAS DWI-positive lesions may need long-term follow-up for cognitive function evaluation.

There were several limitations in our study. First, not every CAS patient routinely received both cerebral MRI and coronary angiography. Even though there was no difference in the demographic data between patients with and without both imaging studies, a selection bias may still be present. Moreover, the study was administered in a single medical institute with limited case numbers, and further recruitment of patients will strengthen the statistical power. Second, the presence of concurrent CAD is the major risk factor for the occurrence of peri-CAS cerebral ischemic lesions in our study, but its underlying mechanism was not well evaluated. Since carotid atherosclerosis has been demonstrated to be associated with the severity of CAD [29], to adopt carotid MRI or ultrasound would be helpful in analyzing the correlation between carotid plaque composition features and risks for peri-CAS cerebral ischemic lesions among patients with varying severity of CAD. Third, the study was conducted in Chinese population with male predominance, and the carotid plaque characteristics would vary among ethnic [30] and gender [31], and the current study results should be verified before further application. Finally, the study mainly focused on the peri-operative neurological complications, and the effect of CAD on the long-term post-CAS neurological manifestations should be monitored in further studies.

## Conclusions

Both symptomatic and asymptomatic CAD can be commonly found in patients with ECAS, and patients with CAD may carry a higher risk for the occurrence of peri-CAS cerebral DWI-positive lesions than patients without CAD. Our study suggests the importance to identify patients with concomitant CAD before undertaking CAS, especially the asymptomatic CAD, and the benefit of CAS should be balanced with the increased risk for peri-operative cerebral ischemic injuries in patients with concomitant CAD.

## Supporting Information

Table S1 **Comparing the clinical data between patients with and without CAD.** The demographic and imaging characteristics of patients with and without CAD.
(DOC)
