# Supplementary material for: Impact of Coexisting Coronary Artery Disease on the Occurrence of Cerebral Ischemic Lesions after Carotid Stenting
Source: PLoS One. 2014 Apr 14;9(4):e94280. doi: 10.1371/journal.pone.0094280 (PMC3986076; doi:10.1371/journal.pone.0094280)
Supplement: Table S1 — Comparing the clinical data between patients with and without CAD. The demographic and imaging characteristics of patients with and without CAD. (DOC) [file pone.0094280.s001.doc]

| **Table S1. Comparing the clinical data between patients with and without CAD.** The demographic and imaging characteristics of patients with and without CAD. | | | |
| --- | --- | --- | --- |
|  | No CAD | With CAD | *P* |
| N | 55 | 71 |  |
| Continuous factors, mean (SD)* |  |  |  |
| Age, years | 67.5 (9.4) | 71.0 (8.4) | 0.0282 |
| Stenosis severity of treated carotid arteries, % | 75.8 (12.0) | 76.3 (10.7) | 0.7987 |
| Stenosis severity of non-treated carotid arteries, % | 33.3 (32.8) | 36.5 (31.5) | 0.5772 |
| Left ventricular ejection fraction, % | 67.0 (8.8) | 62.8 (13.0) | 0.0352 |
| Serum creatinine, mg/dL | 0.95 (0.25) | 1.09 (0.32) | 0.0078 |
| Categorical factors, N (%)^†^ |  |  |  |
| Gender, male | 48 (87) | 64 (90) | 0.6127 |
| Symptomatic carotid artery stenosis | 24 (44) | 23 (32) | 0.1961 |
| Diabetes mellitus | 16 (29) | 30 (42) | 0.1259 |
| Hypertension | 40 (73) | 63 (89) | 0.0212 |
| Dyslipidemia | 38 (69) | 51 (72) | 0.7379 |
| Atrial fibrillation | 3 (5) | 6 (8) | 0.7303 |
| Concomitant vertebral artery stenosis | 26 (47) | 30 (42) | 0.5740 |
| Unilateral | 20 (36) | 21 (30) |  |
| Bilateral | 6 (11) | 9 (12) |  |
| Concomitant iliac artery stenosis | 11 (20) | 17 (24) | 0.5963 |
| Concomitant subclavian artery stenosis | 8 (15) | 8 (11) | 0.5849 |
| Peri-CAS cerebral DWI-positive lesions | 7 (13) | 26 (37) | 0.0018 |
| Peri-CAS manifesting stroke | 1 (2) | 3 (4) | 0.6313 |
| History of anti-platelet medication over 90 days before CAS | 25 (45) | 53 (75) | 0.0008 |
| *Comparison with two-group t test. | | | |
| ^†^Comparison with chi-square test or Fisher’s exact test. | | | |
| CAS, carotid artery stenting ; DWI, diffusion-weighted imaging ; CAD, coronary artery disease. | | | |
